# Broadband long-wave infrared high-absorption of active materials through hybrid plasmonic resonance modes

**DOI:** 10.1186/s11671-023-03817-5

**Published:** 2023-03-08

**Authors:** Xianchao Liu, Zhiheng Zhang, Chao Han, Jiang Wu, Xingchao Zhang, Hongxi Zhou, Qian Xie, Jun Wang

**Affiliations:** 1 School of Optoelectronic Science and Engineering, University of Electronic Science and Technology of China, Chengdu, 610054 China; 2 Institute of Fundamental and Frontier Sciences, University of Electronic Science and Technology of China, Chengdu, 610054 China; 3 Southwest Institute of Technical Physics, Chengdu, 610041 China; 4 State Key Laboratory of Electronic Thin Films and Integrated Devices, Chengdu, 610054 China

**Keywords:** Long-wave infrared, Broadband absorption, Mercury cadmium telluride, Plasmonic resonance, Microelectrode

## Abstract

Broadband high absorption of long-wavelength infrared light for rough submicron active material films is quite challenging to achieve. Unlike conventional infrared detection units, with over three-layer complex structures, a three-layer metamaterial with mercury cadmium telluride (MCT) film sandwiched between an Au cuboid array and Au mirror is studied through theory and simulations. The results show that propagated/localized surface plasmon resonance simultaneously contribute to broadband absorption under the TM wave of the absorber, while the Fabry–Perot (FP) cavity resonance causes absorption of the TE wave. As surface plasmon resonance concentrates most of the TM wave on the MCT film, 74% of the incident light energy is absorbed by the submicron thickness MCT film within the 8–12 μm waveband, which is approximately 10 times than that of the rough same thickness MCT film. In addition, by replacing the Au mirror with Au grating, the FP cavity along the *y*-axis direction was destroyed, and the absorber exhibited excellent polarization-sensitive and incident angle-insensitive properties. For the corresponding conceived metamaterial photodetector, as carrier transit time across the gap between Au cuboid is much less than that of other paths, the Au cuboids simultaneously act as microelectrodes to collect photocarriers generated in the gap. Thus the light absorption and photocarrier collection efficiency are hopefully improved simultaneously. Finally, the density of the Au cuboids is increased by adding the same arranged cuboids perpendicular to the original direction on the top surface or by replacing the cuboids with crisscross, which results in broadband polarization-insensitive high absorption by the absorber.

## Highlights

1. Compared with single-layer MCT film, the submicron same thickness MCT film sandwiched between Au cuboids and Au mirror shows approximately 10 times enhanced absorption (8–12 μm waveband).
2. As the absorption mechanism for TM/TE, wave is propagated/localized surface plasmon resonance and FP cavity resonance, respectively, we can easily achieve polarization absorption by replacing Au mirror with Au grating.
3. For the corresponding conceived photodetector model, Au cuboids are potential to act as microelectrodes to effectively collect photocarriers generated in the gap between Au cuboids, which will improve responsibility and response speed of the photodetector.

## Introduction

Metamaterial absorbers based on surface plasmon resonance (SPR) and plasmonic cavity resonance are quite well known for subwavelength light confinement and localized field improvement [1]. Considering the advantages, metamaterial absorbers have been suggested and successfully used to enhance the light capture of active material layers [2–9]. For the photodetectors, if the absorption efficiency and polarization characteristic could be maintained while decreasing the bulk of active dielectrics, a small active bulk is always preferable because it brings numerous advantages, such as low thermal fluctuation/dark current [10], fast response [11], efficient carrier collection, and low material cost [12–14]. Among all metamaterial absorbers used for this purpose, plasmonic cavities seem to be a good candidate due to their efficient light coupling, easily tunable resonance, and good compatibility with device structures [2–4, 15–20]. However, narrow-band and polarization-insensitive absorption, and ohmic loss (electron scattering) are some major shortcomings of plasmonic devices that limit their applications in this scenario [2, 21]. Metamaterials consisting of a metal mirror, dielectric spacer layer, and complex top metal pattern layers that can excite two or more plasmonic resonances in the operating light waveband have been proposed in recent years to achieve infrared broadband absorption [22–29]. It is worth noting that metamaterials, absorbing based on SPRs and FP cavity resonance, et al., with unit cells much smaller than the working light wavelength, have been proposed and tested during the last few years and show support for potential integration with infrared focal plane arrays [22, 27–29]. The metal mirror simultaneously enhances the light absorption using SPRs and FP cavity resonance, whereas the presence of polarization-insensitive FP cavity resonance in the light wavebands makes it difficult to achieve broadband polarization absorption [22]. Significant effort has been put into searching for methods to circumvent the ohmic loss of metals. However, only a little progress has been made, which includes designing better plasmonic materials with tunable carrier concentration and high mobility [30, 31], developing an effective photonic approach by effectively increasing the volume of the active region, switching the resonance to a higher-order one, and making real facets of the cavity boundaries [32, 33]. Metamaterials with one-dimensional gratings and single-sized cut-wires as a top layer exhibit broadband polarization absorption properties, similar to the metamaterials with small permittivity imaginary part of dielectric spacer [27, 34]. Broadband (polarization) absorption in a simple three-layer structured photodetector is rarely observed. Meanwhile, the developing of active materials growth technologies [35–37], spin-coated quantum materials [38] and experiment progress in lithography craft of active materials insure increasing feasibility of clever combination of active materials and metamaterials [36, 39, 40], while easy-fabrication active materials with unit sizes smaller than the common image element size and pattern feature sizes larger than 400 nm that are compatible with the infrared focal plane are rare and worthy of investigation.

Herein, we propose a long-wave infrared (LWIR) broadband high absorption detection structure with a simple configuration. Polarization-selective absorption, polarization-insensitive absorption, and incident-angle-insensitive absorption are achieved by modifying the configuration of the metamaterial element in theory as well as in simulations. First, we propose an Au cuboid-MCT-Au mirror metamaterial absorber and then analyze the broadband high absorption of the absorber and MCT film. In the next step, the resonance of the TE mode in the FP cavity (electric field direction along the *y*-axis) was meticulously suppressed to achieve broadband polarization-sensitive and incident angle-insensitive absorbers. For the corresponding conceived photodetector, Au cuboids are potential to act as microelectrodes to effectively collect photocarriers generated in the gap between Au cuboids are analyzed. Finally, broadband polarization and an incident angle-insensitive absorber are obtained by adding the same arranged Au cuboids perpendicular to the direction of the original cuboid configuration present in the void area of the top layer, or by replacing the cuboids with crisscross patterns. The average absorption of the absorber and MCT film in a wavelength range of 8–12 μm reaches up to 82% and 74% under the normal incidence of TM waves, respectively. The feature size of the metamaterial turns out to be larger than 400 nm with a period of 4 μm, which is easy to fabricate and compatible with infrared pixels. Broadband absorbers have the potential to improve detector performance.

## Results and discussion

**Fig. 1:**
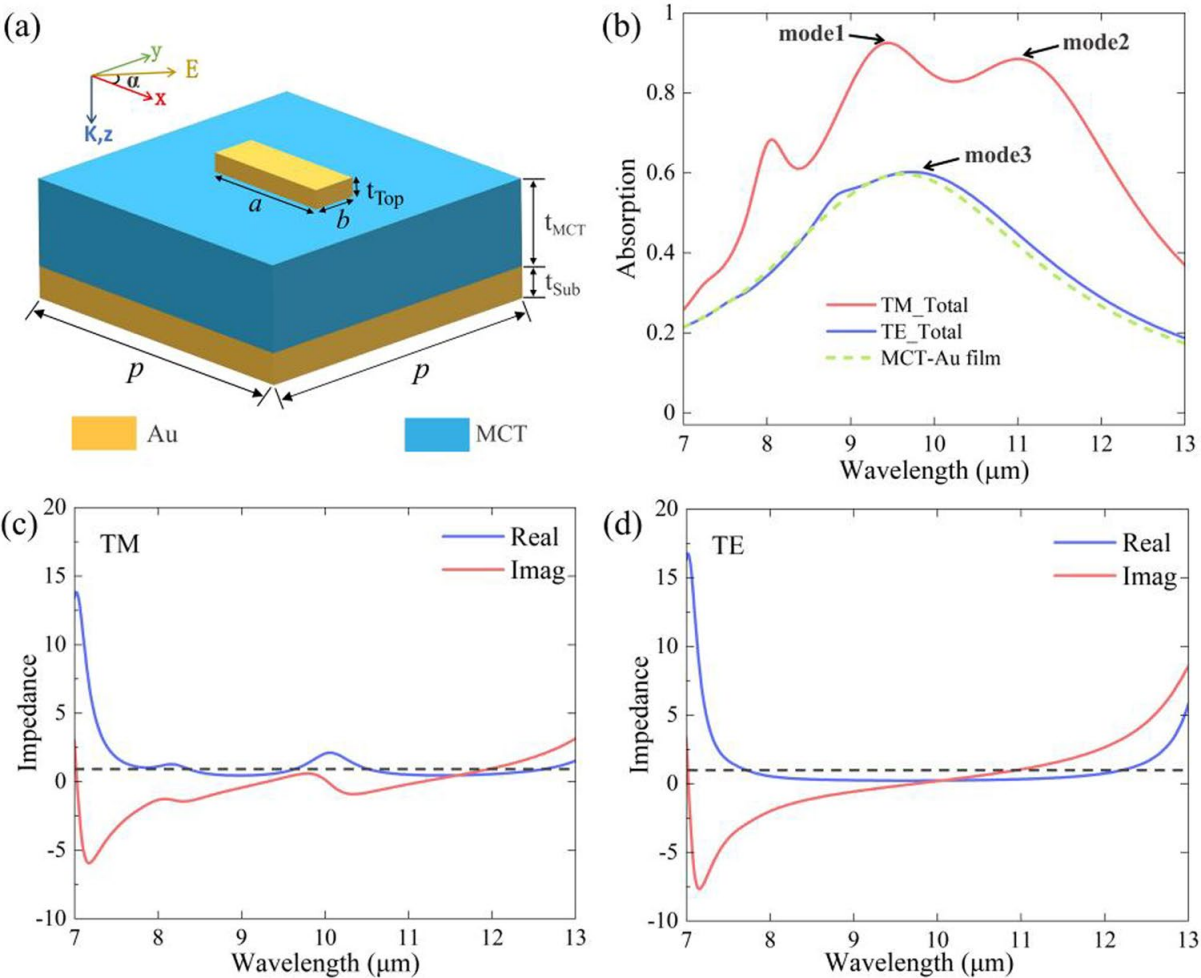
**a** Schematic diagram of the metamaterial element, which consists of a gold bottom and gold cuboid resonator array separated by MCT film. **b** The absorption spectrum of the absorber, the MCT-Au film under TM and TE-waves. **c** The impedance of the absorber for the TM-wave. **d** The impedance of the absorber for the TE-wave. The black dotted line is the air impedance

A polarization-sensitive metamaterial photodetector element was constructed by first proposing a classic model unit consisting of the spacer layer (MCT) sandwiched between the Au cuboid and Au mirror, as shown in Fig. 1. The CST Microwave Studio software was used to build the model and simulate interactions between the incident plane electromagnetic wave and a metamaterial element. The period *P* of both the units along the *x*- and *y*-axes is 4 µm, which is much smaller than the LWIR working wavelength. The length and width of the Au cuboid resonator are *a* (1250 nm) and *b* (400 nm), respectively. The thicknesses of the top layer and spacer are *t*_Top_ and *t*_MCT_. A perfect reflection from the Au mirror was achieved by setting the thickness of the mirror to 150 nm. As proposed, the metamaterial photodetector (mainly the MCT film) showed high absorption within LWIR, which suggests that the thickness of the MCT film should be thicker than that of the top Au cuboid. Optimizing for the conditions, *t*_MCT_ and *t*_Top_ were set to 680 nm and 100 nm, respectively. The value of permittivity reported in Palik [41] was used for the MCT material, which has a tiny fluctuation near *ε*_MCT_ = 13 + *i*0.85 in 7–15 µm waveband. The propagation direction of the plane wave is along the *z*-axis, and the polarization direction makes an angle *α* with the *x*-axis. *S*-parameters (including normalized reflectance, absorptivity, and transmittance) and electric/magnetic field distributions were obtained using CST Microwave Studio software.

As shown in Fig. 1b, normalized absorption under different polarized incident lights was achieved. Broadband absorption for the TM-polarized incident light (electric field direction along the *x*-axis, *α* = 0°) and moderate absorption for the TE-polarized incident light were obtained in 8–12 µm waveband. The average absorption of the TM-and TE-polarized incident light within this waveband was approximately 82% and 50%, respectively. To highlight the mechanism of polarization-dependent absorption, the equivalent impedance of the metamaterial absorber was calculated as follows [42]:

1 $$Z = \sqrt {\frac{{\left( {1 + S_{11} } \right)^{2} - S_{21}^{2} }}{{\left( {1 - S_{11} } \right)^{2} - S_{21}^{2} }}}$$

where *Z* = *Z*′ + *iZ*″. The equivalent impedance was obtained from the scattering parameters (*S*-parameters) and the air impedance was set to *Z*_air_ = 1. According to $R = \frac{{\left( {Z^{\prime} - 1} \right)^{2} + \left( {Z^{\prime\prime}} \right)^{2} }}{{\left( {Z^{\prime} + 1} \right)^{2} + \left( {Z^{\prime\prime}} \right)^{2} }}$, both the real and imaginary parts of the equivalent impedance influence the reflectance of the metamaterial absorber [42]. A near-perfect absorption occurs at the wavelengths where the equivalent impedance of the metamaterial is equal to that of the air. As shown in the real and imaginary parts of the impedance and absorption spectra for the TM-polarized incident light in Fig. 1c and b, the high absorption waveband corresponds to a value near 1 (0) of the impedance real (imaginary) part, which is reasonable (the black dotted line corresponds to the air impedance). However, the impedance for the TE-polarized incident light in Fig. 1d is obviously different from that of air, resulting in a low absorption of the metamaterial in this case.

**Fig. 2:**
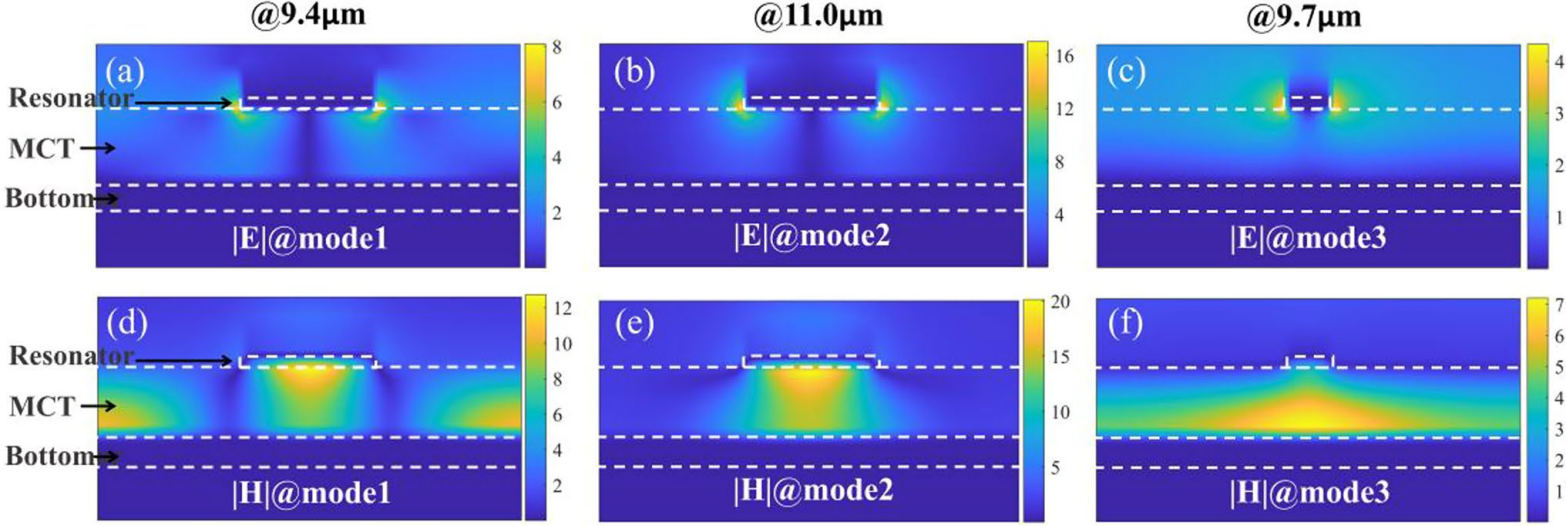
**a** and **d** Electric/magnetic field distribution of *x*-*z* plane at the absorption peak of 9.4 µm. **b** and **e** Electric/magnetic field distribution of *x*-*z* plane at the absorption peak of 11.0 µm. **c** and **f** The electric/magnetic field distributions of *y*-*z* plane at an absorption peak of 9.7 µm, from left to right are mode1, mode2, and mdoe3, respectively

**Fig. 3:**
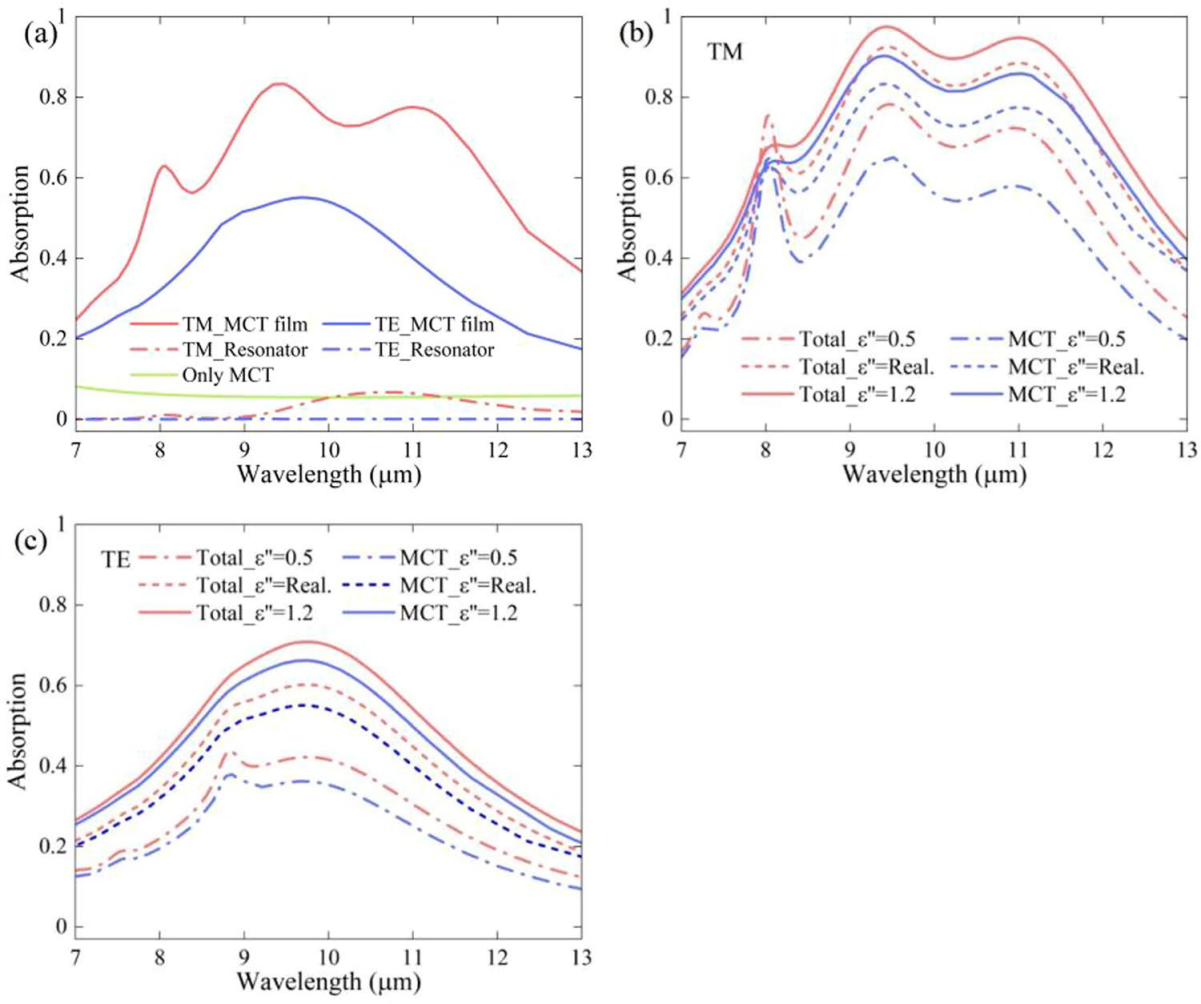
**a** The absorption spectrum of the main layers among the absorbers under the TM wave. **b** The absorption spectrum of the absorber, the MCT film among the absorber with different permittivity imaginary under TM wave. **c** The absorption spectrum of the absorber, the MCT film among the absorber with different permittivity imaginary under TE wave

To explore the reason behind the broadband absorption of TM-waves and the single-peak absorption of TE-waves, the electric and magnetic field distributions at the three main absorption peaks (corresponding to resonance mode1, mode2 and mode3, shown in Fig. 1b) were calculated. As observed in the obtained field distribution (the cross section along the *x*-*z* plane), surface plasmon polariton (SPP) is stimulated at the edge of Au cuboid resonance and results in SPP-induced light capture, shown in Fig. 2a and b (TM wave incidence). An enhanced electric field between the neighboring Au resonances is observed in Fig. 2a and b. In Fig. 2d, the magnetic field is mainly concentrated on the upper and lower surfaces of the resonators, whereas the MCT-Au mirror interface shows the characteristics of the propagated surface plasmon resonance (PSPR, corresponding to mode1 at 9.4 μm). The magnetic field in mode2 (Fig. 2e) is distributed on the upper and lower surfaces of the resonator as well as on the active cavity below the resonator and showed the characteristics of a localized surface plasmon resonance (LSPR, at 11.0 μm), which can be explained by the LC circuit model [43]. Therefore, the broadband absorption of the incident TM-wave by the absorber is observed at least in the hybrid modes of PSPR and LSPR, having a proper resonance peak distance and being excited by the Au resonator array. It is necessary for the metamaterials with an active spacer layer to quantify the incident light energy. Therefore, the absorption spectrum of each layer was calculated based on the following equation [6]:

2 $$Q\left( \omega \right) = \frac{1}{2} \times \omega \times \varepsilon^{\prime\prime} \times \left| {E\left( \omega \right)} \right|^{2}$$

where *ω* is the angular frequency, *ε*″ is the imaginary part of the material permittivity, and *E*(*ω*) is the electric strength corresponding to *ω*. The normalized absorption spectrum of the main layers among the absorber is shown in Fig. 3a. It reveals that the energy of the incident light is mainly absorbed by the MCT film (intrinsic absorption) and the cuboid resonators (also absorbed by the Au mirror). In the 8–12 µm waveband, the average absorption by the MCT film for the incident TM-wave can be as high as 74%, while the average absorption of rough (meaning of stand-alone) MCT film with the same thickness is about 7.0% (the green line in Fig. 3a). The absorption of the former is approximately 10 times stronger than that of the latter. The average absorption of the resonator for the TM wave is only approximately 3% (red dotted line in Fig. 3a), which is much less than the resonator absorption (ohmic loss) reported in the literature [26]. The similar result also occurs for TE-wave case (blue lines in Fig. 3a). The relatively lower TE-wave absorption of MCT film among the absorbers than TM-wave absorption is due to weaker field focusing ability of thin and low-unit-coverage FP cavity. To know the importance of imaginary part value of the MCT permittivity for absorption, we adjusted the imaginary value, and the corresponding absorption of the absorber and the MCT film among the absorbers is shown in Fig. 3b. It is observed that when the imaginary permittivity is 0.5, the average absorption of the MCT film under the TM wave plummets to approximately 53% for 8–12 µm waveband. When the imaginary permittivity is 1.2, the average absorption for the TM wave increases to approximately 79%. Thus, we conclude that the absorption is significantly influenced by the permittivity of the metamaterial. Considering the extremely low absorption of the rough MCT film, we arrive at the conclusion that the excited SPP significantly enhances the intrinsic absorption of the MCT film by concentrating most of the TM incident light on the MCT film.

Unlike the conditions of TM-wave incidence in Fig. 2c and f (mode3, at 9.7 μm), the enhanced electric field is localized near the lateral side as well as below the Au resonance, and the magnetic field is distributed relatively uniformly at the interface of the MCT-Au mirror for the incident TE-wave. This proves that the resonator does not show SPR characteristics in the waveband of interest, and the absorption peak here is the result of the FP cavity mode from the MCT-Au film. This is also evidenced by the absorption spectrum of the MCT-Au film in Fig. 1b, which is nearly the same as that of the absorber for a TE wave. The resonance peak of the MCT-Au film could also be calculated using the reference formula in Ref. [44]. We also calculated the absorption spectra of the absorber and MCT film using different imaginary parts for the MCT material permittivity, shown in Fig. 3c, which indicates that the FP cavity may help to enhance the absorption by providing a relatively uniform concentration of the TM incident light on the MCT film.

**Fig. 4:**
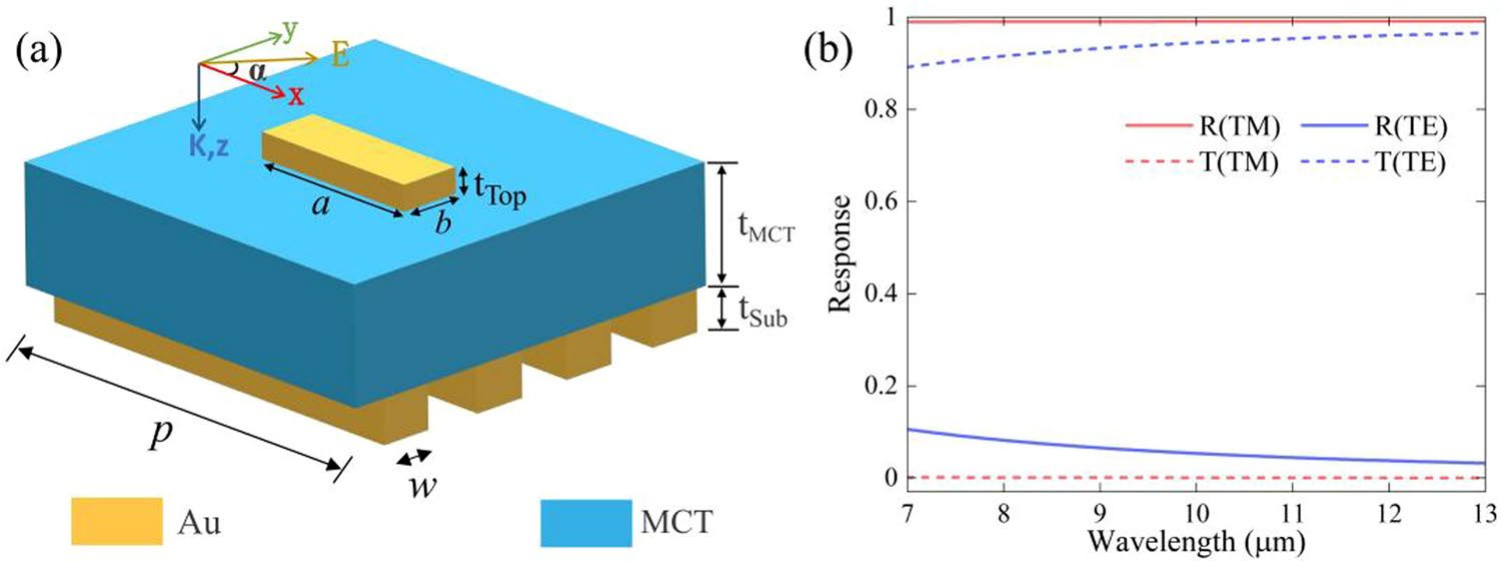
**a** Schematic diagram of the metamaterial element, which consists of gold gratings and gold resonator array separated by an active dielectric layer. **b** The reflective & transmission spectrum of the absorber under TM & TE waves

**Fig. 5:**
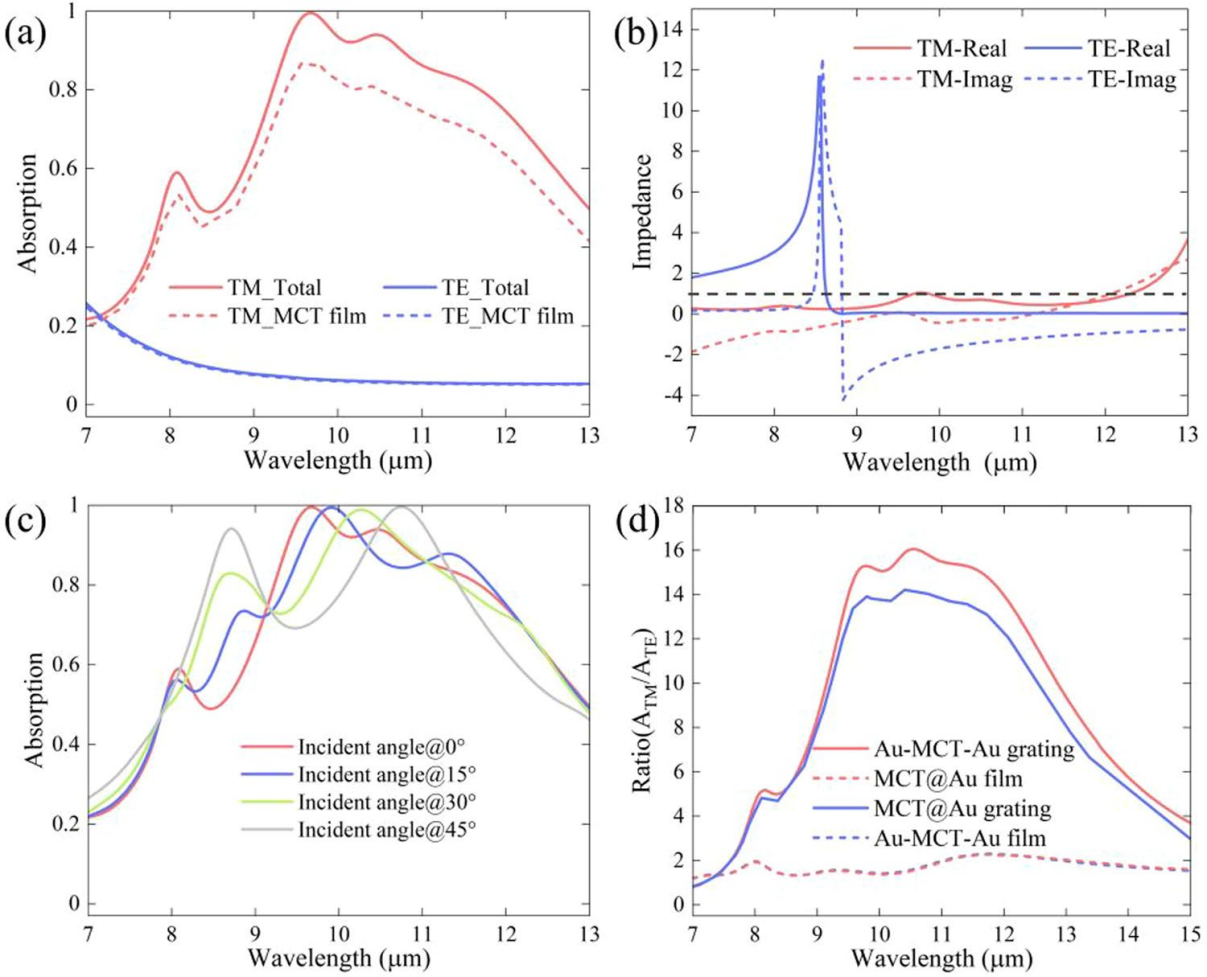
**a** The absorption spectrum of the absorber and MCT film under TM and TE waves. **b** The equivalent impedance of the absorber under TM-wave (red line) and TE-wave (blue line). **c** The absorption spectrum of the absorber under TM-waves. **d** Extinction ratio of absorbers and MCT film among the absorbers with bottom Au film and Au grating, respectively. The black dotted line is the air impedance

Polarization photodetection is required under many conditions. Meanwhile, the incident light energy needs to be concentrated on the active material layer to generate as many photogenerated electrons as possible. By considering simultaneous perfect reflection of TM waves and high transmission of TE waves from one-dimensional Au gratings, better polarization performance is achieved without destroying the existing broadband high absorption of TM wave. A full unit cell with one-dimensional Au grating is shown in Fig. 4a. To ensure that the designed Au grating can meet the requirements, we compared the effect of a pair of gratings (the duty cycle of the grating is 1:1) among one metamaterial element on the reflective (transmission) spectra for the TM (TE) wave. The one-dimensional Au grating with a period of 1000 nm (quarter of the metamaterial unit period) shows perfect reflection of the TM wave and extremely high transmission of the TE wave in the 7–13 µm waveband, as shown in Fig. 4b. Therefore, the Au mirror was replaced by an Au grating with a period of 1000 nm. The absorption spectra of the total metamaterial and the MCT film are shown in Fig. 5a. It is evident that the absorption of the metamaterial absorber and MCT film of the TM wave remains almost the same, while the average absorption of the metamaterial absorber and MCT film of the TE wave is suppressed below 5% in the 7–13 µm waveband. The corresponding impedance of the absorber is shown in Fig. 5b. The impedance of the TM wave shows only small changes, whereas the impedance of the TE wave deviates from the air impedance. These results and their similarities/differences with the impedance of the absorber in Fig. 1c and d can be explained via their S-parameters and the Eq. (1) of equivalent impedance. Figure 5c shows the relation between the absorption spectra and the incident angle of the absorber for the TM wave, which indicates the incident-angle-insensitive property of the absorber. We also investigated the extinction ratio of the absorber and MCT film, as shown in Fig. 5d. The ratio within 9–13 µm is approximately 11.9 for the absorber and 12.7 for the MCT film, which is comparable to the extinction ratio of ordinary-performance commercial polarizers.

In addition, it is worth mentioning that when the suggested absorbers, under LWIR wave illumination, are configured with electrodes atop with appropriate bias for each period, they work as photoconductives. As the electric field distribution in Fig. 2, the photocurrent may mainly generate among the MCT film close to the Au cuboids. The Au cuboids here, with lower resistance than that of MCT and small gaps from adjacent ones, may result that the current flows from one Au cuboid to the MCT in the gap and then to the next Au cuboid [45]. That is, the Au cuboids are also act as microelectrodes to effectively collect photocarriers generated in the gap between Au cuboid because carrier transit time across the gap is much less than that of other paths. Therefore, the LWIR light absorption and photocarrier collection efficiency can be improved simultaneously. The aforementioned properties suggest that the absorber has a lot of potential for usage in polarization-sensitive and angle-insensitive LWIR broadband detection applications.

**Fig. 6:**
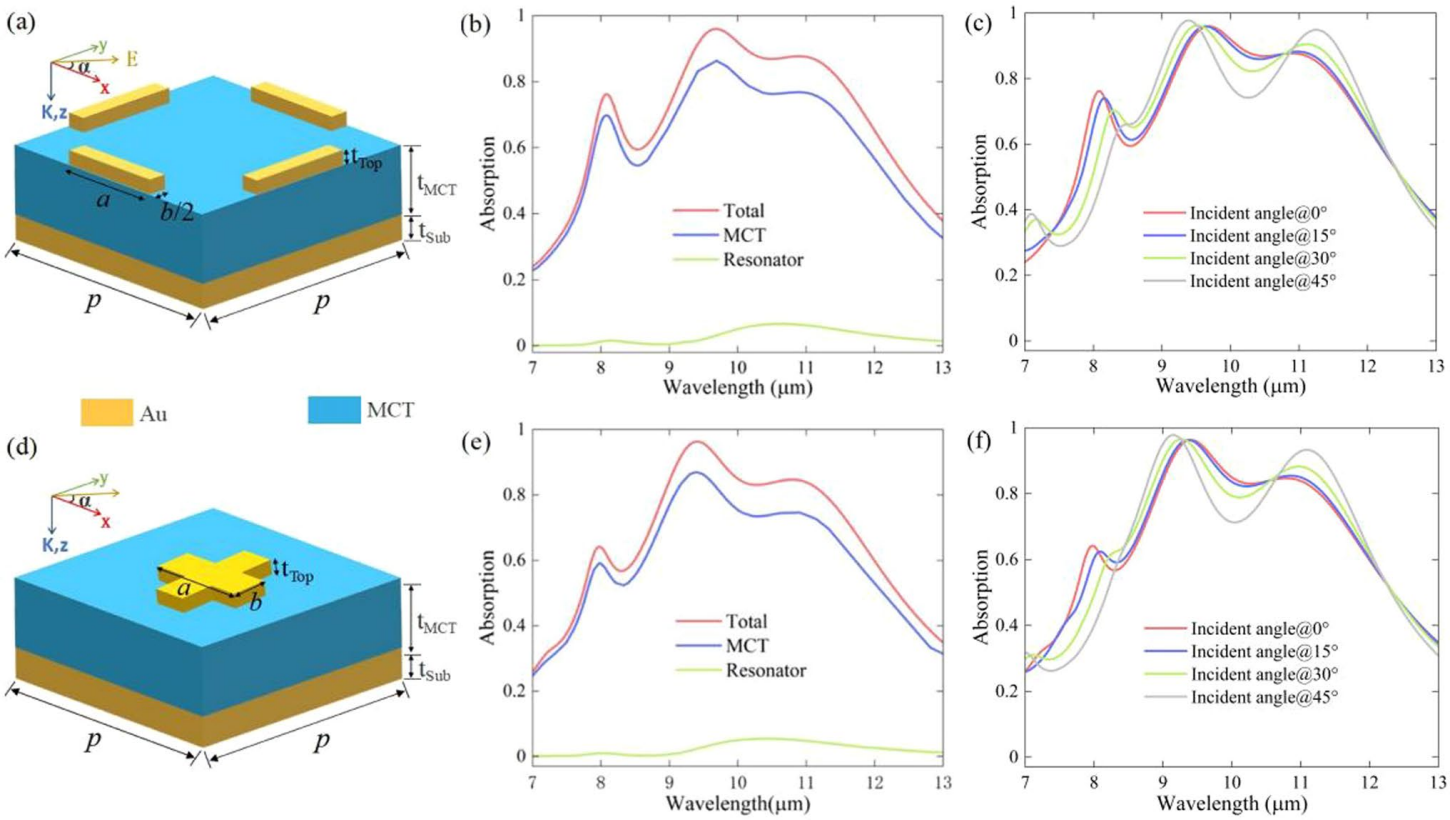
**a**–**c** Schematic diagram of the incident angle-and polarization-insensitive broadband metamaterial element, the corresponding absorption spectrum of the metamaterial absorber and each layer of the absorber, and the dependence on the incident angle and absorption spectrum of the element. **d**–**f** Schematic diagram of the incident angle-and polarization-insensitive broadband metamaterial element, the corresponding absorption spectrum of the absorber and each layer of the absorber, and the dependence on the incident angle and absorption spectrum of the absorb

In a few applications, such as cloaks and microbolometers, polarization-insensitive and incident-angle-insensitive absorptions are required. Thus, we added another Au cuboid array with the same configuration as the top layer at the void area, which was perpendicular to the existing cuboid array, as shown in Fig. 6a. The new absorber has high symmetry, and thus, its absorption spectrum for the TM and TE waves is the same, showing broadband high absorption in the 8–12 µm waveband. The same broadband high absorption was also obtained for the TM and TE waves (shown in Fig. 6e), when a symmetrical cross-resonator array, as shown in Fig. 6d, was designed by rotating the Au cuboid array. We also studied the relationship between absorption and the light incident angle on the absorbers in Fig. 6 and found incident angle-insensitive properties of the absorbers, as shown in Fig. 6c and f. The discussed results have proved that the MCT sandwiched between cuboid resonators and the Au mirror is an effective way to achieve high performance of polarization sensitive/insensitive detection for LWIR.

## Summary

In conclusion, polarization-dependent and polarization-independent broadband high absorption submicron thickness metamaterial photodetectors were demonstrated in theory and simulation by using a composite active metamaterial design. The broadband, high absorption of the TM incident light in the 8–12 µm waveband resulted from a simultaneous PSPR and LSPR response to the incident light and provided increased opportunities for intrinsic absorption of the MCT film. The absorption of the MCT film was observed to be as high as 74% of the total incident TM light energy. Absorption of the TE incident light was due to the FP cavity resonance. Replacement of the Au mirror with a grating with the same orientation as the long side improved the extinction ratio of the broadband polarized absorber. Besides enhancing light absorption of the metamaterial, the Au cuboids atop the metamaterial photodetector are also potential to act as microelectrodes to improve photocarriers collection efficiency. Finally, the modified absorber with the long side of the Au cuboids along *x* and *y* directions, or with cross-Au resonators, showed polarization-insensitive spatially concentrated broadband absorption and incident angle-insensitive absorption. The designed metamaterial absorber can be used in hot-electron devices, infrared imaging, thermal detection, and other devices.

## Data Availability

All datasets are presented in the main paper and be freely available to any scientist wishing to use them for non-commercial purposes, without breaching participant confidentiality.

## Abbreviations

MCT: Mercury cadmium telluride
FP: Fabry–Perot
SPR: Surface plasmon resonance
LWIR: Long-wave infrared
SPP: Surface plasmon polariton
PSPR: Propagated surface plasmon resonance
LSPR: Localized surface plasmon resonance

## References

[1] Luo XG (2019). Subwavelength artificial structures: opening a new era for engineering optics. Adv Mater.

[2] Palaferri D, Todorov Y, Bigioli A, Mottaghizadeh A, Gacemi D, Calabrese A, Vasanelli A, Li L, Davies AG, Linfield EH, Kapsalidis F, Beck M, Faist J, Sirtori C (2018). Room-temperature nine-μm-wavelength photodetectors and GHz-frequency heterodyne receivers. Nature.

[3] Zhou Y, Li ZF, Zhou X, Zhou J, Zheng Y, Li L, Li N, Chen PP, Chen XS, Lu W (2019). Cut-off wavelength manipulation of pixel-level plasmonic microcavity for long wavelength infrared detection. Appl Phys Lett.

[4] Jing YL, Li ZF, Li Q, Chen XS, Chen PP, Wang H, Yao M, Li N, Lu W (2016). Pixel-level plasmonic microcavity infrared photodetector. Sci Rep.

[5] Knight MW, Sobhani H, Nordlander P, Halas NJ (2011). Photodetection with active optical antennas. Science.

[6] Li W, Valentine J (2014). Metamaterial perfect absorber based hot electron photodetection. Nano Lett.

[7] Miyazaki HT, Mano T, Kasaya T, Osato H, Watanabe K, Sugimoto Y, Kawazu T, Arai Y, Shigetou A, Ochiai T, Jimba Y, Miyazaki H (2020). Synchronously wired infrared antennas for resonant single-quantum-well photodetection up to room temperature. Nat Commun.

[8] Jing YL, Li ZF, Li Q, Chen PP, Zhou XH, Wang H, Li N, Lu W (2016). Angular dependence of optical modes in metal-insulator-metal coupled quantum well infrared photodetector. AIP Adv.

[9] Wang D, Allcca AEL, Chung TF, Kildishev AV, Chen YP, Boltasseva A, Shalaev VM (2020). Enhancing the graphene photocurrent using surface plasmons and a p-n junction. Light Sci Appl.

[10] Burghartz JN (2013). Guide to state-of-the-art electron devices.

[11] Assefa S, Xia F, Vlasov YA (2010). Reinventing germanium avalanche photodetector for nanophotonic on-chip optical interconnects. Nature.

[12] Lin Q, Armin A, Burn PL, Meredith P (2015). Filterless narrowband visible photodetectors. Nat Photonics.

[13] Han C, Han XW, Han JY, He MY, Peng SL, Zhang CY, Liu XC, Gou J, Wang J (2022). Light-stimulated synaptic transistor with high PPF feature for artificial visual perception system application. Adv Func Mater.

[14] Han JY, Wang FK, Han S, Deng WJ, Du XY, Yu H, Gou J, Wang QJ, Wang J (2022). Recent progress in 2D inorganic/organic charge transfer heterojunction photodetectors. Adv Func Mater.

[15] Dixon K, Montazeri AO, Shayegannia M, Barnard ES, Cabrini S, Matsuura N, Holman HY, Kherani NP (2020). Tunable rainbow light trapping in ultrathin resonator arrays. Light Sci Appl.

[16] Hoque A, Islam MT, Almutairi AF, Faruque MRI (2019). Design of split hexagonal patch array shaped nano-metaabsorber with ultra-wideband absorption for visible and UV spectrum application. Nanoscale Res Lett.

[17] Lochbaum A, Dorodnyy A, Koch U, Koepfli SM, Volk S, Fedoryshyn Y, Wood V, Leuthold J (2020). Compact mid-infrared gas sensing enabled by an all-metamaterial design. Nano Lett.

[18] Liu X, Starr T, Starr AF, Padilla WJ (2010). Infrared spatial and frequency selective metamaterial with near-unity absorbance. Phys Rev Lett.

[19] Long XY, Yue W, Su YR, Chen WD, Li L (2019). Large-scale, bandwidth-adjustable, visible absorbers by evaporation and annealing process. Nanoscale Res Lett.

[20] Xu HX, Hu G, Wang Y, Wang C, Wang M, Wang S, Huang YJ, Genevet P, Huang W, Qiu CW (2021). Polarization-insensitive 3D conformal-skin metasurface cloak. Light Sci Appl.

[21] Zhu YS, Xu HX, Yu P, Wang ZM (2021). Engineering plasmonic hot carrier dynamics toward efficient photodetection. Appl Phys Rev.

[22] Zhou Y, Qin Z, Liang ZZ, Meng DJ, Xu H, Smith DR, Liu Y (2021). Ultra-broadband metamaterial absorbers from long to very long infrared regime. Light Sci Appl.

[23] Sang T, Qi H, Wang X, Yin X, Li GQ, Niu XS, Ma B, Jiao HF (2020). Ultrabroadband absorption enhancement via hybridization of localized and propagating surface plasmons. Nanomaterials.

[24] Hendrickson JR, Vangala S, Dass C, Gibson R, Goldsmith J, Leedy K, Walker DE, Cleary JW, Kim W, Guo J (2018). Coupling of epsilon-near-zero mode to gap plasmon mode for flat-top wideband perfect light absorption. ACS Photonics.

[25] Jiang ZH, Yun S, Toor F, Werner DH, Mayer TS (2011). Conformal dual-band near-perfectly absorbing mid-infrared metamaterial coating. ACS Nano.

[26] Parsamyan H, Haroyan H, Nerkararyan K (2022). Broadband tunable mid-infrared absorber based on conductive strip-like meta-atom elements. Mater Today Commun.

[27] Qin Z, Meng DJ, Yang FM, Shi XY, Liang ZZ, Xu HY, Smith DR, Liu YC (2021). Broadband long-wave infrared metamaterial absorber based on single-sized cut-wire resonators. Opt Express.

[28] Kim T, Bae YJ, Lee N, Cho HH (2019). Hierarchical metamaterials for multispectral camouflage of infrared and microwaves. Adv Func Mater.

[29] Sun K, Vassos E, Yan X, Wheeler C, Churm J, Wiecha PR, Gregory SA, Feresidis A, de Groot CH, Muskens OL (2022). Wafer-scale 200 mm metal oxide infrared metasurface with tailored differential emissivity response in the atmospheric windows. Adv Opt Mater.

[30] Khurgin JB (2015). How to deal with the loss in plasmonics and metamaterials. Nat Nanotechnol.

[31] Zheng K, Song J, Qu J (2018). Hybrid low-permittivity slot-rib plasmonic waveguide based on monolayer two dimensional transition metal dichalcogenide with ultra-high energy confinement. Opt Express.

[32] Zhen T, Zhou J, Li ZF, Chen XS (2019). Realization of both high absorption of active materials and low ohmic loss in plasmonic cavities. Adv Opt Mater.

[33] Le Perchec J, Desieres Y, Espiau de Lamaestre R (2009). Plasmon-based photosensors comprising a very thin semiconducting region. Appl Phys Lett.

[34] Hao J, Yuan Y, Ran L, Jiang T, Kong JA, Chan CT, Zhou L (2007). Manipulating electromagnetic wave polarizations by anisotropic metamaterials. Phys Rev Lett.

[35] Shen Z, Deng Z, Zhao X, Huang J, Yao L, Zhou X, Cao C, Gao Q, Chen B (2020). Long-wave infrared sub-monolayer quantum dot quantum cascade photodetector. J Lightwave Technol.

[36] Wang L, Xu Z, Xu J, Dong F, Wang F, Bai Z, Zhou Y, Chai X, Li H, Ding R, Chen J, He L (2020). Fabrication and characterization of InAs/GaSb type-II superlattice long-wavelength infrared detectors aiming high temperature sensitivity. J Lightwave Technol.

[37] Venkatasamy V, Jayaraju N (2006). Formation of HgCdTe by electrochemical atomic layer epitaxy (EC-ALE). ECS Trans.

[38] Chen W, Deng Z, Guo D, Chen Y, Mazur YI, Maidaniuk Y, Benamara M, Salamo GJ, Liu H, Wu J, Chen B (2018). Demonstration of InAs/InGaAs/GaAs quantum dots-in-a-well mid-wave infrared photodetectors grown on silicon substrate. J Lightwave Technol.

[39] Nordin L, Petluru P, Kamboj A, Muhowski AJ, Wasserman D (2021). Ultra-thin plasmonic detectors. Optica.

[40] Lindquist NC, Luhman WA, Oh SH, Holmes RJ (2008). Plasmonic nanocavity arrays for enhanced efficiency in organic photovoltaic cells. Appl Phys Lett.

[41] Palik ED (1991). Handbook of optical constants of solids ii.

[42] Tittl A, Harats MG, Walter R, Yin X, Schäferling M, Liu N, Rapaport R, Giessen H (2014). Quantitative angle-resolved small-spot reflectance measurements on plasmonic perfect absorbers: impedance matching and disorder effects. ACS Nano.

[43] Ye YQ, Jin Y, He S (2010). Omnidirectional, polarization-insensitive and broadband thin absorber in the terahertz regime. J Opt Soc Am B.

[44] ElKabbash M, Iram S, Letsou T, Hinczewski M, Strangi G (2018). Designer perfect light absorption using ultrathin lossless dielectrics on absorptive substrates. Adv Opt Mater.

[45] Yao Y, Shankar R, Rauter P, Song Y, Kong J, LoncarM CF (2014). High-responsivity mid-infrared graphene detectors with antenna-enhanced photocarrier generation and collection. Nano Lett.

